# Correlation between Inflammasomes and Microbiota in Peri-Implantitis

**DOI:** 10.3390/ijms25020961

**Published:** 2024-01-12

**Authors:** Miguel Padial-Molina, Saray Montalvo-Acosta, Natividad Martín-Morales, Virginia Pérez-Carrasco, Antonio Magan-Fernandez, Francisco Mesa, Francisco O’Valle, Jose Antonio Garcia-Salcedo, Pablo Galindo-Moreno

**Affiliations:** 1 Department of Oral Surgery and Implant Dentistry, School of Dentistry, University of Granada, 18071 Granada, Spain; mipadial@ugr.es; 2 Instituto de Investigación Biosanitaria ibs.GRANADA, 18012 Granada, Spain; 3 PhD Program in Clinical Medicine and Public Health, University of Granada, 18071 Granada, Spain; 4 PhD Program in Biomedicine, University of Granada, 18071 Granada, Spain; 5 Department of Pathology, School of Medicine, University of Granada, 18071 Granada, Spain; 6 Centre for Genomics and Oncological Research, Pfizer–University of Granada–Andalusian Regional Government (GENYO), PTS Granada, 18016 Granada, Spain; 7 Microbiology Unit, University Hospital Virgen de las Nieves, 18014 Granada, Spain; 8 Department of Periodontics, School of Dentistry, University of Granada, 18071 Granada, Spainfmesa@ugr.es (F.M.); 9 Institute of Biopathology and Regenerative Medicine (IBIMER, CIBM), University of Granada, 18071 Granada, Spain

**Keywords:** peri-implantitis, inflammasome, NLRP3, AIM2, caspase-1, IL-1β, microbiome

## Abstract

The activation of inflammasomes is thought to induce the inflammatory process around dental implants. No information is available on the correlation between microbiota and inflammasomes in clinical samples from patients suffering peri-implantitis. For this cross-sectional study, 30 biofilm samples were obtained from 19 patients undergoing surgical treatment for peri-implantitis because of the presence of bleeding on probing, probing depth higher than 6 mm, and radiographic bone loss higher than 3 mm. Then, soft tissue samples from around the implant were also collected. The relative abundance of bacteria and alpha-diversity indexes were calculated after analyzing the 16S rRNA gene using next-generation sequencing. The soft-tissue samples were processed for evaluation of the inflammasomes NLRP3 and AIM2 as well as caspase-1 and IL-1β. The relative abundance (mean (SD)) of specific species indicated that the most abundant species were *Porphyromonas gingivalis* (10.95 (14.17)%), *Fusobacterium vincentii* (10.93 (13.18)%), *Porphyromonas endodontalis* (5.89 (7.23)%), *Prevotella oris* (3.88 (4.94)%), *Treponema denticola* (2.91 (3.19)%), and *Tannerella forsythia* (2.84 (4.15)%). Several correlations were found between the species and the immunohistochemical detection of the inflammasomes NLRP3 and AIM2 as well as caspase-1 and IL-1β, both in the epithelium and the lamina propria. A network analysis found an important cluster of variables formed by NLRP3 in the lamina propria and AIM2, caspase-1, and IL-1β in the lamina propria and the epithelium with *Prevotella dentalis*, *Prevotella tannerae*, *Tannerella forsythia*, or *Selenomonas timonae*. Thus, it could be concluded that inflammasomes NLRP3 and AIM2 and their downstream effectors caspase-1 and interleukin-1β can be significantly associated with specific bacteria.

## 1. Introduction

Peri-implantitis, as defined, is “a plaque-associated pathological condition…characterized by inflammation in the peri-implant mucosa” [1]. This inflammation in the soft tissue ends up affecting the surrounding bone, which leads to the destruction of the supportive peri-implant structures. To date, no successful therapy is available. In fact, the literature, comparatively, still indicates the same methods as 10 years ago both for cleaning and decontamination of the implant surface [2,3] and for reconstructing the tissues lost [4,5]. Thus, better understanding of the microbial–host interactions and additional factors must be achieved.

A fundamental step differentiating a reversible peri-implant disease (i.e., mucositis) from the state of bone destruction (i.e., peri-implantitis) is the increase in interleukin-1β (IL-1β) that disrupts the osteoclast–osteoblast balance [6]. Within the context of periodontal and peri-implant diseases, that the production of IL-1β mediated by caspase-1 is a consequence of the activation of molecular complexes known as inflammasomes has been recently proposed [7]. Inflammasomes can be activated in response to pathogen-associated molecular patterns (PAMPs) such as the interferon-inducible protein absent in melanoma 2 (AIM2). Other types of inflammasomes, such as the nucleotide-binding oligomerization domain-like receptors family pyrin domain containing 3 (NLRP3), are activated by both PAMPs and damage-associated molecular patterns (DAMPs) [6]. Thus, the evaluation of the whole picture and of both pathways separately is important. Particularly, our group has recently published for the first time in the literature a characterization of the expression of both AIM2 and NLRP3 in peri-implantitis lesions from human patients. That study demonstrates that both inflammasomes are increased in peri-implantitis and correlate with other markers of inflammation, tissue destruction, and clinical variables such as probing pocket depth (PPD) [8].

Non-bacterial-associated damage may originate from cement from the prosthesis. However, in the case of screw-retained implant connections, the only source of this type of DAMPs can be the release of debris from the friction between the two components. The role that implant debris, such as titanium particles and ions, may play in both osseointegration itself and in the initiation of peri-implant marginal bone resorption is being highly debated currently in dental implantology [9,10,11,12]. While debris from the implant itself may be released to the peri-implant bone, such debris may also be produced at the interface between the prosthesis and the implant or between the implant and the transmucosal abutments [13]. In fact, numerous studies have found titanium particles released from the implants in the surrounding tissues [14,15,16,17,18,19,20,21]. Within this debate, the activation of NLRP3, specifically, may help in finding a biologically plausible explanation for the differences between periodontitis and peri-implantitis. Thus, it is being studied, particularly in vitro [22]. Our group has also recently confirmed the activation in vitro of NLRP3 in response to titanium ions and of AIM2 in response to lipopolysaccharides (LPSs); interestingly, when both titanium and LPSs are combined, NLRP3 is predominant, while AIM2 decreases [23]. In fact, the presence of titanium in the peri-implant sulcus has been associated with a modification of the structure and a reduction in the diversity of the microbiome associated with peri-implant health and disease [24]. However, an analysis of the complex microbial communities present in clinical samples and how they correlate with the expression of inflammasomes is also required but, to our knowledge, has not been studied yet.

Thus, the current study aims to evaluate the microbial profiles in samples obtained from peri-implantitis lesions and correlate the findings with the characterization of the inflammatory process, particularly with respect to the expression of the inflammasomes AIM2 and NLRP3 as well as caspase-1 and interleukin-1β.

## 2. Results

### 2.1. Clinical Variables

The current study evaluated a set of 30 samples from implants with peri-implantitis obtained from a total of 19 patients. Table 1 shows the clinical data both at the patient level and at the implant level. In summary, most of the patients included were female (63.16%), nonsmokers (52.63%), and did not consume alcohol regularly (89.47%). At the implant level, confirming the diagnosis, all implants had biofilm attached, presented bleeding on probing, and had around 7 mm of probing in all sites after removal of the prosthetic restoration. Most of the implants were located in the posterior mandible (50.00%), supported fixed metal-ceramic bridges (80.00%), and had been in function for more than 10 years (60.00%). In this study, 56.67% of the implants were Microdent^®^ (Ti grade 4, sandblasted surface), 36.67% were AstraTech TX^®^ (Ti grade 4, Osseospeed^®^ surface treatment), and 6.67% were type Branemark MK system (Ti grade 4, machined surface).

### 2.2. Immunohistochemical Results

**Figure 1:**
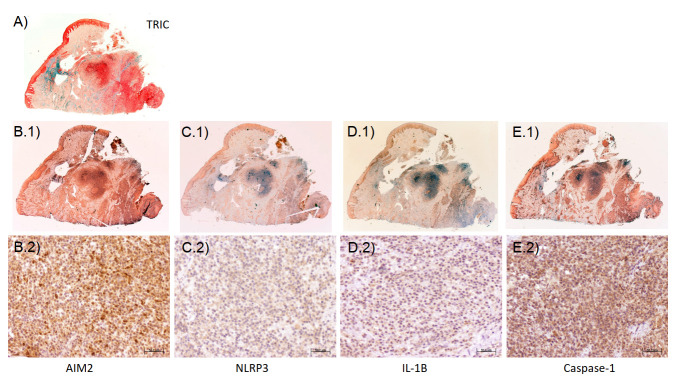
Representative immunohistochemical images of a section stained with (**A**) Masson trichrome (original magnification 1.25×) and to detect the expression of inflammasome components (**B.1**,**B.2**) AIM2 and (**C.1**,**C.2**) NLRP3 as well as (**D.1**,**D.2**) IL-1β and (**E.1**,**E.2**) caspase-1 (original magnification at (**1**) 1.25× and (**2**) 20×). Peroxidase-conjugated micropolymer method visualized with diaminobenzidine.

Table 2 summarizes the immunohistochemical expression of the inflammasomes AIM2 and NLRP3 as well as caspase-1 and IL-1β. Representative images of the immunohistochemical expression of AIM2, NLRP3, caspase-1, and IL-1β are presented in Figure 1. There are significant correlations between the clinical variables, such as average probing depth and the expression of inflammasomes, as well as between the expression of AIM2, NLRP3, caspase-1, and IL-1β (Supplementary Table S1).

### 2.3. Microbial Analysis

The sequencing of the 16S rRNA gene amplicon libraries with the Illumina MiSeq platform gave a total of 372,270 sequences after bioinformatic processing (an average of 12,409 reads per sample). To minimize the sample-size-induced bias among the samples, all were rarefied by sub-sampling at 2000 sequence reads. This sequencing depth made it possible to obtain a coverage higher than 95% in all samples. Extraction and amplification controls were also amplified and sequenced, showing no significant amplification and a smaller number of sequences.

**Figure 2:**
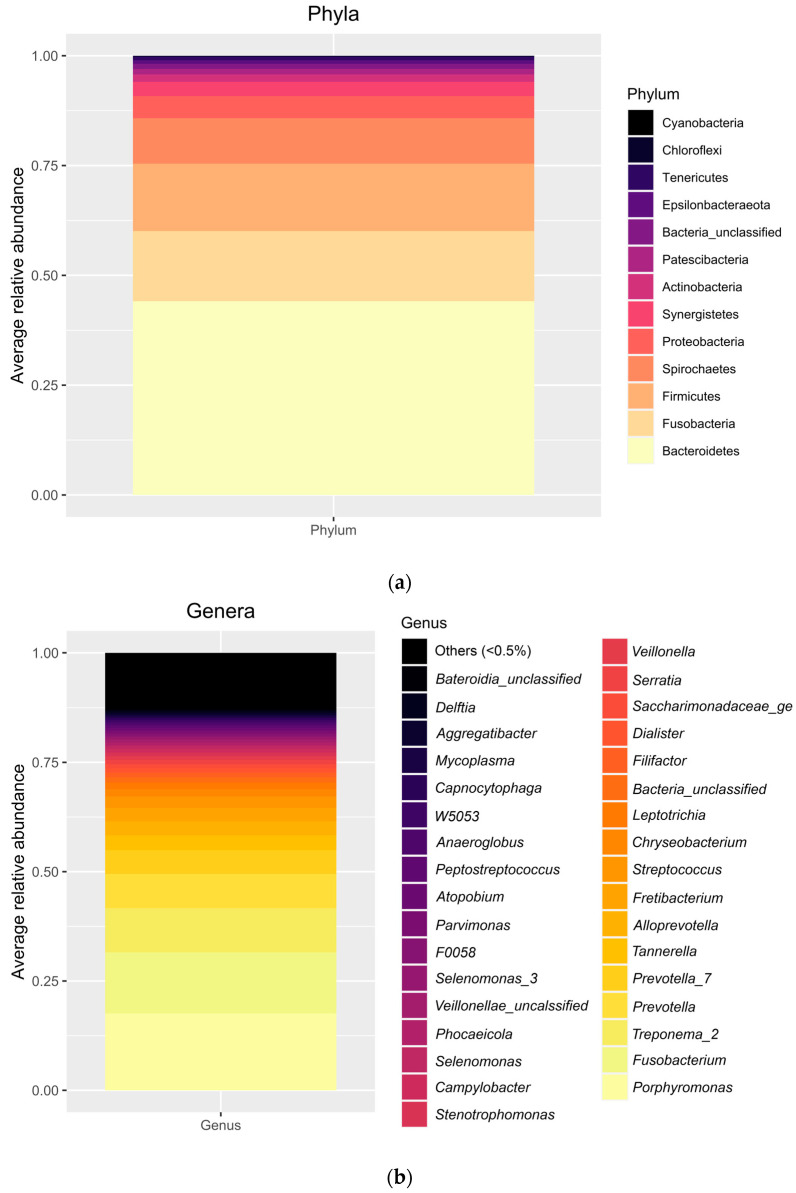

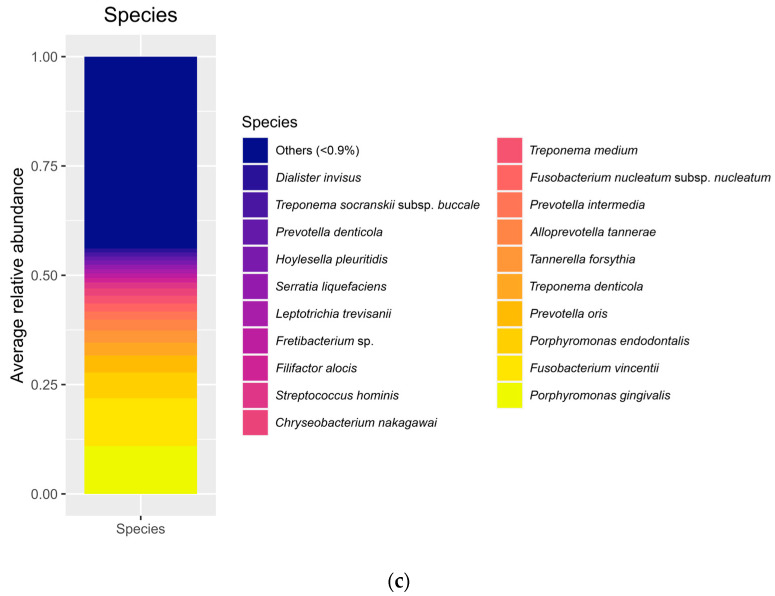
(**a**) Relative abundance at the phylum level in the overall population evaluated. Four phyla are not depicted, as their relative abundance was below 0.001%. (**b**) Relative abundance at the genus level in the overall population evaluated. Note that genera with an overall relative abundance below 0.5% are represented combined (12.72%). (**c**) Relative abundance at the OTU level in the overall population evaluated. Note that OTUs with an overall relative abundance below 0.9% are represented combined (43.87%).

A total of 17 phyla were detected; only 10 of them had a relative abundance above 1%. As expected, in all individual samples evaluated the more abundant phylum was Bacteroidetes, followed by Firmicutes, Fusobacteria, and Spirochaetes. In average, Bacteroidetes represented 44.12 (12.25)%, Fusobacteria 15.98 (14.55)%, Firmicutes 15.31 (8.74)%, and Spirochaetes 10.36 (10.35)% of all the phyla. Other, less abundant phyla, below 5%, included Proteobacteria, Synergistetes, Actinobacteria, Patescibacteria, and unclassified bacteria. Figure 2a represents the relative abundance at the phylum level in each individual sample and in the overall population evaluated. At the genus level (Figure 2b), a total of 343 genera were detected. The most abundant were *Porphyromonas* (17.56 (14.63)%) followed by *Fusobacterium* (14.00 (14.69)%), *Treponema_2* (10.08 (10.48)%), *Prevotella* (7.86 (9.12)%), *Prevotella_7* (5.44 4.68)%), and *Tannerella* (3.32 (4.41)%). As for the level of species (Figure 2c), the most abundant species were *Porphyromonas gingivalis* (10.95 (14.17)%), *Fusobacterium vincentii* (10.93 (13.18)%), *Porphyromonas endodontalis* (5.89 (7.23)%), *Prevotella oris* (3.88 (4.94)%), *Treponema denticola* (2.91 (3.19)%), and *Tannerella forsythia* (2.84 (4.15)%).

Regarding the alpha-diversity of the microbial populations, the different indexes are shown in Supplementary Table S2. Chao1, an abundance-based estimator of species richness, shows an estimation of approximately 715 OTUs in our samples. The high value of the Shannon index (>3) and the high value of the inverse Simpson (or the low value, 0.09, close to zero, of the Simpson index) indicate a high bacterial diversity. The Pielou index, a measure of the species evenness, shows that the species are not evenly distributed in the bacterial community, but that there are more enriched species than others in the community.

### 2.4. Correlations between Clinical, Histological, Immunohistochemical, and Microbial Variables

The inverse Simpson, Shannon, and Pielou indexes were significantly correlated with both age and average probing depth, so that the higher the age or the probing depth, the lower the diversity or evenness index (Table 3). At the phylum level, significant correlations were found only between age and Synergistetes and between tobacco consumption and Tenericutes (Table 4). When the analysis evaluated the correlations at the genus level, several correlations were found with age, gender, and alcohol and tobacco consumption. Significant correlations were also found between average probing depth and the genera *Mycoplasma* (Spearman’s rho = 0.510; *p* = 0.004; 95% CI: 0.173, 0.740) and *Centipeda* (Spearman’s rho = −0.370; *p* = 0.044; 95% CI: −0.651, 0.000) (Table 5). Additional data are available in the Supplementary Spreadsheet.

Regarding the histological data on the extension of the inflammatory infiltrate, no significant correlation was found with any of the calculated indexes nor with any microbiological data at the level of phyla. Significant negative correlations were found only between the extension of the inflammatory infiltrate and the genus *Bacteroides* (Spearman’s rho = −0.368; *p* = 0.045; 95% CI: −0.649, 0.002) (Table 6).

The immunohistochemical expression in specific locations of some inflammasome components and IL-1β were significantly correlated with several phyla (Table 7). Briefly, AIM2 in the lamina propria was correlated with the phylum Synergistetes, NLRP3 in the lamina propria was correlated with Synergistetes and Tenericutes, and IL-1β in the epithelium was correlated with Epsilonbacteraeota. As with the clinical data, we detected several correlations between the immunohistochemical expression in specific locations of some inflammasome components as well as caspase-1 and IL-1β and several genera of bacteria. These data are available in the Supplementary Spreadsheet.

At the species level, several correlations were found between microbial data and age, alcohol consumption, and tobacco habits, as well as with average probing depth (Table 8). Particularly, average probing depth was positively correlated with the amount of *Porphyromonas gingivalis*, *Campylobacter mucosalis*, *Metamycoplasma orale*, *Mogibacterium timidum*, and *Metamycoplasma salivarium*, while the correlation was negative with *Fusobacterium polymorphum*. Similarly, several correlations were found between microbial data at the level of species and the immunohistochemical detection of the inflammasomes NLRP3 and AIM2 as well as caspase-1 and IL-1β, both in the epithelium and the lamina propria (Table 9).

### 2.5. Network Analysis

**Figure 3:**
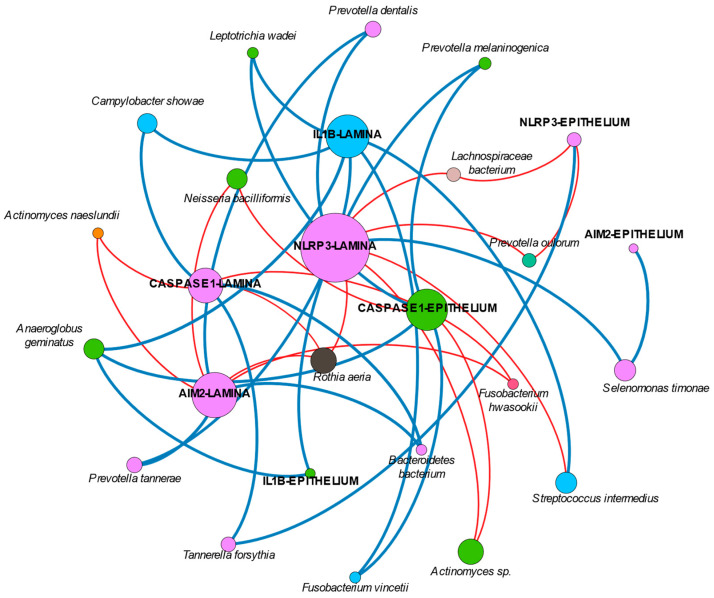
Graphical representation of the network analysis. Note that only those significant correlations above 0.4 or below −0.4 are represented in the graph. The color of the lines indicates whether the correlation was positive (blue line) or negative (red line); the color of the nodes represents different clusters.

The network analysis found several correlations between the different variables and permitted us to generate several clusters of variables highly related between them (Figure 3). In summary, an important cluster of variables is formed by the nodes representing the NLRP3 expression in the lamina propria, which is connected to the expression of AIM2, caspase-1, and IL-1β in the lamina propria and the epithelium, either directly or through an intermediate interaction with *Prevotella dentalis*, *Prevotella tannerae*, *Tannerella forsythia*, or *Selenomonas timonae*. *Rothia aeria* is an important connector negatively associated with NLRP3, AIM2, and caspase-1 in the lamina propria. A different cluster includes the expression of caspase-1 in the epithelium with the species *Anaeroglobus geminatus*, *Actinomyces sp.*, *Neisseria bacilliformis,* and *Prevotella melaninogenica*. Finally, there is another cluster formed between the expression of IL-1β in the lamina propria and *Campylobacter showae*, *Streptococcus intermedius,* and *Fusobacterium vincentii*. Most of these clusters include nodes positively correlated between them. The Supplementary Data spreadsheet contains all the significant correlations between the different species and the immunohistochemical data.

## 3. Discussion

The current study was aimed at analyzing the correlation between the abundance of specific microbes detectable in peri-implantitis sulci and the expression of the inflammasomes AIM2 and NLRP3 as well as caspase-1 and interleukin-1β. Our findings demonstrate significant negative and positive correlations between them.

The relative abundance of several bacteria found in our samples represents the classical ecology of oral environments, and, particularly, of peri-implantitis lesions as described previously by a number of studies [25,26,27,28]. Specifically, the diversity indices can be considered high, in line with previous studies [29,30,31]. Interestingly, the literature is consistent in reporting a decrease in diversity as the disease evolves [32]. In contrast, we have found a negative correlation between several diversity indexes and the average probing depth, which would be more in line with recent theories about the understanding of peri-implantitis as a dysbiosis condition based on the loss of microbial diversity and the loss of keystone taxa that could favor the bloom of pathogens and changes in metabolic activities [33].

Because of the absence of similar correlation studies between the variables of interest in the current analysis, we can only compare with those reporting on the presence or absence of microorganisms. According to this, it can be said that the phyla found in our study to be significantly correlated with inflammasomes in the lamina and the epithelium of peri-implantitis lesions (mainly Synergistetes and Tenericutes) had been previously associated with the severity of peri-implantitis lesions. In this sense, old studies such as that from 2008 by Shibli et al. using DNA-DNA checkerboard or that from 2010 by Koyanagi et al. using 16S rRNA gene analysis detected Synergistetes and Tenericutes only in peri-implantitis but not in periodontitis nor in healthy implants [25,34]. Most recent studies have found similar results [35,36,37]. The positive correlation with the level of NLRP3 in the lamina propria we found in the current study may indicate a confirmation that these phyla play a role in the development of the disease.

Regarding the other significantly associated phylum, Epsilonbacteraeota, it must be said that very limited information has been published about it in the periodontal literature [38]. Nothing has been found regarding its role in peri-implantitis. In fact, its role is mainly still unknown [38]. Thus, our finding regarding its association with the levels of caspase-1 is speculative only.

Inflammasomes have been recently introduced in the peri-implant literature. As described earlier, both AIM2 and NLRP3 result in the activation of pro-caspase-1, which ultimately activates pro-IL-1β. Thus, the mechanisms by which this activation occurs might be of high interest, not only from a mechanistic point of view, but also as potential therapeutic targets [39]. In fact, it has been described that NLRP3 specifically can play a determinant role in the impairment of fibroblasts’ migration capacities [40] and alveolar bone loss. If suppressed, both age-related [41] and ligature-induced [42] periodontitis-like alveolar bone loss is decreased. Thus, the levels of NLRP3 in saliva have been proposed as markers of periodontal disease [43].

If we take what is known in periodontitis and translate it to peri-implantitis, it is reasonable to think that similar effects may happen. However, although AIM2 would be activated by the same mechanisms in the periodontal and the peri-implant sulci, NLRP3 can be activated by bacteria, bacteria byproducts, and titanium [22,44]. Thus, there would be another activator in peri-implantitis that is not present in periodontitis. Even more, precisely because of the presence of the implant, the peri-implant niche must be considered to be very different compared with the periodontal niche; in fact, it has been described as a “unique microenvironment that force microbial adaptation and selection” [45]. This is because, as recently proposed, titanium products, particularly ions, have the potential to change the microbiological composition of biofilms, specifically reducing their diversity [24,46]. The presence of titanium in the sulcus fluid [14,17,18,24] and the tissue of peri-implantitis lesions has been reported in several studies [21]. Small particles (not visible in optical microscopy), even at the level of just titanium ions, are more frequently released as the consequence of wearing between the prosthesis and the implant; they have been identified in several studies as the most deleterious, as discussed in Wu et al. [47]. Thus, the presence of titanium in the peri-implant sulcus would contribute to a microbial dysbiosis that, together with the titanium ions themselves, may ultimately end up in the development of peri-implantitis. Both inflammasome pathways would function: the pathway that responds to other biological stimuli only, such as AIM2, and the pathway that responds to both biological and physical products, such as NLRP3. This double path for pro-inflammatory inductors could explain the higher severity that is observed in peri-implantitis lesions compared with periodontitis.

However, very little is known so far about inflammasomes in peri-implantitis. Only a recent study by our group has demonstrated that both the inflammasomes AIM2 and NLRP3 are highly expressed in peri-implantitis lesions from human patients and correlate with other markers of inflammation, tissue destruction, and clinical variables such as PD [8]. As expected, similar results regarding those correlations have also been found in the set of samples used in the current study. Moreover, in vitro, when both titanium and LPS are combined, NLRP3 is predominant while AIM2 decreases [23].

The current study found several correlations between different species and clinical and immunohistochemical variables. First, like many other studies in the literature, we found a positive correlation between the average probing depth and the abundance of *Porphyromonas gingivalis*. It was reported that *Porphyromonas gingivalis* may reduce the expression of NLRP3 and IL-1β but not modify the expression of AIM2 in vitro, which would help dampen the host’s innate immune response [48]. In contrast, *A. actinomycetemcomitans* can increase the expression of NLRP3, while again keeping unaltered the expression of AIM2 [49]. In a way, these results come to reinforce the findings of the current study, indicating that inflammasome activation may be increased or decreased depending on the bacteria in action. Our study, clinically and beyond the limitations of the single- or 10-species biofilm models like those executed in the referenced studies, correlates different species with both increased and decreased inflammasome activation. The specific mechanisms by which those bacteria influence the expression of inflammasomes need further investigation.

Interestingly, our network analysis found an isolated node of *Rothia aeria* that is negatively correlated with the expression of NLRP3, AIM2, and caspase-1 in the lamina propria. Many studies have reported negative correlations between *Rothia* and probing depth and disease [28,37,50]. Our findings could be hypothesized as the explanation for those findings.

Finally, the available studies on IL-1β would have to be re-considered in the near future. As recently discussed, levels of IL-1β may suffer post-transcriptional modifications through inflammasomes that may not have been studied appropriately [51]. Thus, differences in health and disease might be not properly represented, particularly if the expression of upstream regulatory molecules, predominantly NLRP3 in peri-implantitis as shown, are not looked at. The role of titanium might be a game-changer in this issue. In fact, if we consider the network analysis, it can be suggested that in peri-implantitis, NLRP3 plays a more relevant role compared with AIM2. In this sense, the association of NLRP3, IL-1β, and several genera has been found to be more dominant than those other clusters formed by AIM2 or caspase-1 and their respective bacteria. However, the current study did not evaluate the presence of titanium in the peri-implant sulcus nor in the lesion tissue, which would have added important and interesting information. In addition, we did not evaluate smoker patients separately nor exclude them from the current study, although tobacco consumption may influence both inflammation and biofilm composition. This is because the sample size was limited, as no previous data were available to propose a valid sample size calculation; so, a subgroups comparison would have reduced the number of patients per group even more. We did not exclude them from the study because smoking is common in patients with peri-implantitis; so, excluding them would have reduced the generalizability of the study. Furthermore, the evaluation of inflammasomes in peri-implantitis tissue has previously demonstrated no correlation with tobacco consumption [8].

We must also acknowledge that this study evaluated samples from a clinical condition only and few markers of the inflammatory pathway, particularly related to the inflammasomes AIM2 and NLRP3. This information was obtained with immunohistochemical techniques. Furthermore, it was only a cross-sectional study, which misses the dynamics of a real clinical environment; a prospective study could provide interesting information on this topic, not only microbiologically but also from the inflammasome activation-deactivation dynamic. Even more, regardless of the remarkable association between bacteria and specific markers of the inflammasome complexes NLRP3 and AIM2 and their locations, we must acknowledge, as highlighted by Belibasakis et al., that current understanding of diseases initiated or modified by bacteria must be understood as community-driven interactions rather than a single type of bacteria orchestrating the whole complex [32]. However, we truly believe that the information provided in this study is highly novel and would be helpful for future studies on the topic. Particularly, it would be interesting to keep the focus on the pathways evaluated here when evaluating more complex functional interactions between the host and the microbial community [52]. Clinically, once more, patients and professionals must be aware of the complexity of the disease and the importance of attending regular maintenance visits to minimize any unbalance that may be initiated and result in peri-implant tissue destruction.

## 4. Materials and Methods

### 4.1. Study Design, Primary Locations, and Participants

In this study, microbial samples obtained from patients with peri-implantitis were analyzed. In addition, mucosa samples had also been obtained during surgical treatment of the disease. These samples were evaluated for both histological and immunohistochemical characterization of the inflammatory and tissue destruction processes, as well as for mRNA expression of the genes of interest. The population for the current study was taken from a pool of samples described in a larger comparative study recently published [8]. All procedures performed were in accordance with the 1964 Helsinki declaration and its later amendments. The protocol of the study was approved by the Ethics Committee for Human Research of the University of Granada (587/CEIH/2020). This study follows the recommendations contained in the STROBE guideline for the reporting of observational studies.

In summary, for this cross-sectional study, patients treated for peri-implantitis at a faculty clinic of the Department of Oral Surgery and Implant Dentistry, School of Dentistry of the University of Granada were asked to participate. To be included in the study, after explaining all the study procedures, they signed an informed consent form. They had to be older than 18, without active periodontal disease, and systemically healthy. With respect to the peri-implant disease, the diagnosis of peri-implantitis followed the 2017 World Workshop on the Classification of Periodontal and Peri-implant Diseases and Conditions [53]: presence of bleeding on probing, probing depth higher than 6 mm, and radiographic bone loss higher than 3 mm that required surgical therapy. The implants suffering peri-implantitis had to support a screw-retained prosthesis, be in normal occlusal function for more than 1 year, and not treated during the previous 6 months. All patients were included from June 2020 through July 2021. On the other hand, patients were excluded if they had been under antibiotic and anti-inflammatory therapy at any time during the previous 3 months, suffered any systemic disease or uncontrolled diabetes, if the prosthesis could not be removed for any reason, or if the implant was mobile.

### 4.2. Outcomes Measures

#### 4.2.1. Clinical Data

Data were registered regarding patients’ age, gender, alcohol and tobacco consumption, and, at the implant level, the presence of biofilm and probing pocket depth (PPD) and bleeding on probing (BOP) in four sites around the implant, after removal of the prosthesis, using a periodontal probe.

#### 4.2.2. Immunohistochemical Analysis

Immunohistochemical techniques were used to visualize the expression, location, and number of positive cells per mm^2^ for each of the inflammasome multiprotein complexes (AIM2 and NLRP3) as well as caspase-1 and IL-1β. In summary, after collection of the microbial samples and recording of the clinical data, resective surgical therapy was conducted under local anesthesia [54]. A band of soft tissue was obtained from the vestibule/lingual aspect of the lesion through an internal bevel incision that extended apically beyond the bottom of the peri-implant sulcus. After collecting the samples, they were fixed in 10% formaldehyde for 48 h at room temperature, after which they were paraffin-embedded and sectioned. Then, sections in the slides were deparaffinized and processed for immunohistochemical staining through rehydration and treatment in a pretreatment thermal PT module (Thermo Fisher Scientific Inc., Waltham, MA, USA) with a 1 mM EDTA buffer pH 8, for 20 min at 95 °C. After blocking with peroxidase for 15 min and washing with a TBS+Tween20 buffer, the polyclonal primary antibodies anti-NLRP3 (1:400 dilution), anti-caspase-1 (1:4000 dilution), anti-interleukin 1β (1:400 dilution) (Thermo Fisher Scientific Inc.), and anti-AIM2 (1:200 dilution) (Abcam, Cambridge, UK), were applied for 30 min at room temperature. The slides were processed in an automatic immunostainer (Autostainer480S, Thermo Fisher Scientific Inc.) for visualization of the staining using a peroxidase-conjugated micropolymer and the chromogen diaminobenzidine. After that, Mayer’s progressive hematoxylin stain was used as contrast.

Following the previously described methodology, immunohistochemical expression was evaluated by quantifying the number of positive cells per mm² in 10 fields per section using a BH2 microscope (Olympus Optical Company, Ltd., Tokyo, Japan) with a 40× objective and a millimeter scale in the eyepiece.

#### 4.2.3. Microbial Analysis

The day of the surgery, before probing or removal of the prosthesis, peri-implant plaque samples were obtained from the sulcular space of the peri-implant pocket. Briefly, excess saliva was removed with an ejector and the area was isolated with cotton rolls. Then, biofilm was collected by inserting a sterile paper tip into the pocket for 30 s. The area (lingual or vestibular) was selected according to the area where the tissue sample was going to be collected; this was defined in a previous visit when the diagnosis and treatment plan was established. The paper strip was then transferred to an Eppendorf tube and frozen at −80 °C.

Bacterial DNA extraction was performed as previously described [55] with some modifications. Briefly, paper tips containing the sample were dissolved in 100 μL of lysis buffer (3% *w*/*v* sodium dodecyl sulphate in 50 mM tris, 5 mM EDTA, pH 8.0, 10 μg/mL RNase A) at 68 °C for one hour; then, the mixture was recovered and transferred to a sterile tube, and the stated procedure followed from this point. Negative controls were included in each extraction batch to ensure the absence of contaminants. DNA integrity and quality were evaluated and then quantified using a spectrophotometer (NanoDrop 2000 UV–Vis; Thermo Fisher Scientific Inc.).

PCR amplification products of the V1–V3 variable regions of the 16S rRNA gene were obtained using fusion universal primers 27F (Illumina adaptors + 5′AGAGTTTGATCMTGGCTCAG3′) and 533R (Illumina adaptors + 5′TTACCGCGGCKGCTGGCACG3′), as previously described [55,56,57]. Similarly, negative controls were included in each PCR batch to check for the absence of contaminants. Amplicon multiplexing and sequencing was performed with a dual indexing tag-tailed design using 8 nt indices from the Nextera XT Index Kit v2 (Illumina, San Diego, CA, USA). Paired-end sequencing of 16S PCR amplicon libraries was performed using the Illumina MiSeq instrument with v3 kit chemistry (300 + 300 bp). Bioinformatics analysis and quality filtering were performed using Mothur software v1.43.0 (University of Michigan Medical School, Ann Arbor, MI, USA), following the standard MiSeq operating procedure. Chimeric reads were identified and excluded using Chimera UCHIME. Redundant, nonchimeric FASTA files were taxonomically classified using Silva v132 database. Alpha-diversity was examined with operational taxonomic units (OTUs) at 3% dissimilarity and the distance-based greedy clustering (DGC) algorithm, calculating the Chao1, inverse Simpson, Shannon, and Pielou diversity indices. The most representative sequence of each OTU was extracted, and this sequence was used as input in BLAST [58] to identify the corresponding bacterial species.

#### 4.2.4. Statistical Analysis

Statistical analyses were performed using nonparametric tests with the SPSS 28.0 software for macOS (IBM Inc., Chicago, IL, USA), Microsoft Excel 16.71 for macOS (Microsoft^®^ Corporation, Redmond, WA, USA), and R software (version 4.3.2, R Foundation for Statistical Computing, Vienna, Austria). The relative abundance of each phylum, genus, and species was calculated within each sample. Mean (standard deviation) and percentages were used for continuous and categorical variables, respectively. Only genera with total abundance >0.01% were considered for statistical analysis. Correlations between clinical, histological, and microbial variables were evaluated using the Spearman’s rho correlation coefficient. In all cases, a *p* < 0.05 was considered to represent statistical significance.

Additionally, a network analysis was conducted to determine the possible association of groups of variables. Briefly, the immunohistochemical expressions of AIM2, NLRP3, caspase-1, and IL-1β, both in the lamina propria and in the epithelium, were evaluated together with the microbial data at the species level. Network diagram was represented using Gephi v.0.9.2 with cut-off values of −0.4 and 0.4, including only significant correlations (*p* < 0.05).

## 5. Conclusions

Results from the current study indicate that the inflammasomes NLRP3 and AIM2, and their downstream effectors caspase-1 and interleukin-1β, can be significantly associated with specific bacteria at the level of phyla and genera. This suggests potential specific interactions that need further investigation.

## Figures and Tables

**Table 1:** Clinical data of patients evaluated in the current study.

**Patient-Level Variables (n = 19)**	
Age (mean (min–max) in years)	60 (48–75)
Gender (n (%))	
Female	12 (63.16)
Male	7 (36.84)
Alcohol (n (%))	
No	17 (89.47)
<10 g per day	2 (10.53)
Smokers (n (%))	
No	10 (52.63)
<10 cigarettes per day	9 (47.37)
**Implant-Level Variables (n = 30)**	
Type of implant (n (%))	
Branemark MK system (Ti grade 4, machined surface)	2 (6.67)
AstraTech TX^®^ (Ti grade 4, Osseospeed^®^ surface treatment)	11 (36.67)
Microdent^®^ (Ti grade 4, sandblasted surface)	17 (56.67)
Time in function (n (%))	
1–5 years	4 (13.33)
6–10 years	8 (26.67)
>10 years	18 (60.00)
Type of prosthesis (n (%))	
Fixed (single)	1 (3.33)
Fixed (bridge)	24 (80.00)
Hybrid	2 (6.67)
Overdenture	3 (10.00)
Implant position (n (%))	
Posterior maxilla	5 (16.67)
Anterior maxilla	2 (6.67)
Posterior mandible	15 (50.00)
Anterior mandible	8 (26.67)
Biofilm (n (%))	
No	0 (0.00)
Yes	30 (100.00)
Bleeding on probing (n (%))	
No	0 (0.00)
Yes	30 (100.00)
Probing Depth (mean (SD) in mm)	
Average	6.93 (2.52)
Mesial	6.80 (2.62)
Distal	7.37 (2.97)
Lingual	6.63 (3.03)
Vestibular	6.93 (2.82)

**Table 2:** Immunohistochemical expression of inflammasomes AIM2 and NLRP3 as well as caspase-1 and interleukin-1β.

Marker	Zone	Mean of Positive Cells per mm^2^ (SD)
AIM2	Lamina propria	3204.67 (1278.70)
	Epithelium	642.94 (450.45)
NLRP3	Lamina propria	3200.99 (1109.27)
	Epithelium	1431.61 (817.71)
Caspase-1	Lamina propria	2698.73 (1445.68)
	Epithelium	1001.79 (612.86)
Interleukin-1β	Lamina propria	1997.64 (1252.82)
	Epithelium	646.51 (602.97)

**Table 3:** Correlations between clinical data and indexes. Only significant correlations are shown.

Variable	Index	Spearman’s Rho	*p* Value	95% CI
Age	Inverse Simpson	−0.422	0.020	−0.685, −0.062
	Shannon	−0.487	0.006	−0.726, −0.142
	Pielou	−0.410	0.025	−0.677, −0.047
Average probing depth	Inverse Simpson	−0.394	0.031	−0.667, −0.028
	Shannon	−0.377	0.040	−0.656, −0.008

**Table 4:** Correlations between clinical data and phylum. Only significant correlations are shown.

Variable	Phylum	Spearman’s Rho	*p* Value	95% CI
Age	Synergistetes	−0.418	0.022	−0.682, −0.057
Tobacco	Tenericutes	0.681	< 0.001	0.416, 0.839

**Table 5:** Correlations between clinical data and genus. Only significant correlations are shown.

Variable	Genus	Spearman’s Rho	*p* Value	95% CI
Average probing depth	*Mycoplasma*	0.510	0.004	0.173, 0.740
	*Centipeda*	−0.370	0.044	−0.651, 0.000

**Table 6:** Correlations between histological data and genus. Only significant correlations are shown.

Variable	Genus	Spearman’s Rho	*p* Value	95% CI
Extension of inflammatory infiltrate	*Bacteroides*	−0.368	0.045	−0.649, 0.002

**Table 7:** Correlations between immunohistochemical data with phyla. Only significant correlations are shown.

Marker and Location	Phylum	Spearman’s Rho	*p* Value	95% CI
AIM2 in lamina propria	Synergistetes	0.402	0.031	0.030, 0.676
NLRP3 in lamina propria	Synergistetes	0.526	0.006	0.162, 0.764
	Tenericutes	0.754	<0.001	0.510, 0.886
IL-1β in epithelium	Epsilonbacteraeota	0.506	0.012	0.116, 0.761

**Table 8:** Correlations between clinical data and species. Only significant correlations are shown.

Clinical Variable	Species	Spearman’s Rho	*p* Value	95% CI
Age	*Prevotella oris*	0.403	0.027	0.039, 0.673
	*Porphyromonas endodontalis*	−0.516	0.004	−0.744, −0.180
	*Aminobacterium colombiense*	−0.365	0.047	−0.648, 0.006
	*Treponema socranskii* subsp. *buccale*	0.405	0.026	0.041, 0.674
	*Aggregatibacter aphrophilus*	−0.372	0.043	−0.652, −0.002
	*Fretibacterium* sp.	−0.403	0.027	−0.673, −0.039
	*Streptococcus constellatus*	−0.361	0.050	−0.645, 0.010
	*Prevotella baroniae*	−0.362	0.049	−0.646, 0.009
	*Treponema vincentii*	−0.409	0.025	−0.676, −0.046
	*Alistipes communis*	−0.412	0.024	−0.679, −0.050
	*Prevotella illustrans*	−0.473	0.008	−0.717, −0.125
	*Saccharimonas aalborgensis*	−0.367	0.046	−0.649, 0.004
	*Prevotella oris*	0.467	0.009	0.117, 0.714
	*Prevotella illustrans*	−0.572	0.001	−0.778, −0.257
	*Treponema maltophilum*	−0.362	0.049	−0.646, 0.009
	*Haemophilus parahaemolyticus*	−0.434	0.017	−0.693, −0.076
Alcohol	*Hoylesella pleuritidis*	0.375	0.041	0.006, 0.654
	*Campylobacter mucosalis*	0.419	0.021	0.058, 0.683
	*Treponema medium*	0.438	0.015	0.081, 0.695
	*Anaeroglobus geminatus*	0.388	0.034	0.022, 0.663
	*Prevotella koreensis*	0.403	0.027	0.038, 0.672
	*Treponema maltophilum*	0.552	0.002	0.229, 0.766
Tobacco	*Aminobacterium colombiense*	0.479	0.007	0.133, 0.721
	*Campylobacter mucosalis*	0.467	0.009	0.117, 0.713
	*Streptococcus constellatus*	0.527	0.003	0.194, 0.750
	*Metamycoplasma orale*	0.523	0.003	0.190, 0.748
	*Saccharimonas aalborgensis*	−0.407	0.026	−0.675, −0.043
	*Prevotella koreensis*	0.475	0.008	0.128, 0.719
	*Metamycoplasma salivarium*	0.423	0.020	0.063, 0.686
	*Catonella massiliensis*	0.480	0.007	0.134, 0.722
	*Treponema maltophilum*	0.530	0.003	0.199, 0.753
	*Prevotellamassilia timonensis*	0.400	0.029	0.035, 0.671
	*Aminobacterium colombiense*	0.405	0.026	0.042, 0.674
Average probing depth	*Porphyromonas gingivalis*	0.456	0.011	0.103, 0.707
	*Fusobacterium polymorphum*	−0.509	0.004	−0.740, −0.172
	*Campylobacter mucosalis*	0.437	0.016	0.080, 0.695
	*Metamycoplasma orale*	0.375	0.041	0.006, 0.654
	*Mogibacterium timidum*	0.370	0.044	0.001, 0.651
	*Metamycoplasma salivarium*	0.422	0.020	0.062, 0.685

**Table 9:** Correlations between histological and immunohistochemical data and species. Only significant correlations are shown.

Immunohistochemical Marker and Location	Species	Spearman’s Rho	*p* Value	95% CI
AIM2 in epithelium	*Treponema pectinovorum*	0.467	0.009	0.117, 0.714
AIM2 in lamina propria	*Aminobacterium colombiense*	−0.572	0.001	−0.778, −0.257
	*Mogibacterium timidum*	−0.362	0.049	−0.646, 0.009
	*Pyramidobacter piscolens*	−0.434	0.017	−0.693, −0.076
NLRP3 in epithelium	*Tannerella forsythia*	0.403	0.027	0.039, 0.673
NLRP3 in lamina propria	*Leptotrichia trevisanii*	−0.516	0.004	−0.744, −0.180
	*Stenotrophomonas maltophilia*	−0.365	0.047	−0.648, 0.006
	*Aminobacterium colombiense*	0.405	0.026	0.041, 0.674
	*Desulfobulbus oralis*	−0.372	0.043	−0.652, −0.002
	*Prevotella dentalis*	−0.403	0.027	−0.673, −0.039
	*Prevotella koreensis*	−0.361	0.050	−0.645, 0.010
	*Metamycoplasma orale*	−0.362	0.049	−0.646, 0.009
	*Sphaerochaeta halotolerans*	−0.409	0.025	−0.676, −0.046
	*Mogibacterium timidum*	−0.412	0.024	−0.679, −0.050
	*Campylobacter mucosalis*	−0.473	0.008	−0.717, −0.125
	*Prevotella illustrans*	−0.367	0.046	−0.649, 0.004
Caspase-1 in epithelium	*Parvimonas micra*	0.375	0.041	0.006, 0.654
	*Campylobacter gracilis*	0.419	0.021	0.058, 0.683
Caspase-1 in lamina propria	*Porphyromonas endodontalis*	0.438	0.015	0.081, 0.695
	*Aminobacterium colombiense*	0.388	0.034	0.022, 0.663
	*Fusobacterium polymorphum*	0.403	0.027	0.038, 0.672
	*Aggregatibacter aphrophilus*	0.552	0.002	0.229, 0.766
	*Prevotellamassilia timonensis*	0.479	0.007	0.133, 0.721
	*Prevotella dentalis*	0.467	0.009	0.117, 0.713
	*Treponema socranskii* subsp. *buccale*	0.527	0.003	0.194, 0.750
IL-1β in epithelium	*Tannerella forsythia*	0.523	0.003	0.190, 0.748
	*Serratia liquefaciens*	−0.407	0.026	−0.675, −0.043
	*Pseudoramibacter alactolyticus*	0.475	0.008	0.128, 0.719
	*Treponema medium*	0.423	0.020	0.063, 0.686
	*Lacticaseibacillus paracasei* subsp. *paracasei*	0.480	0.007	0.134, 0.722
	*Sphaerochaeta halotolerans*	0.530	0.003	0.199, 0.753
	*Campylobacter mucosalis*	0.400	0.029	0.035, 0.671
	*Corynebacterium neomassiliense*	0.405	0.026	0.042, 0.674
IL-1β in lamina propria	*Prevotella oris*	0.456	0.011	0.103, 0.707
	*Parvimonas micra*	−0.509	0.004	−0.740, −0.172
	*Treponema socranskii* subsp. *buccale*	0.375	0.041	0.006, 0.654

## Data Availability

Data supporting the findings presented in this study are available from authors upon reasonable request.

## Supplementary Materials

The supporting information can be downloaded at: https://www.mdpi.com/article/10.3390/ijms25020961/s1.
