# Supplementary material for: Correlation between Inflammasomes and Microbiota in Peri-Implantitis
Source: Int J Mol Sci. 2024 Jan 12;25(2):961. doi: 10.3390/ijms25020961 (PMC10815557; doi:10.3390/ijms25020961)
Supplement: Supplementary file 1 [file ijms-25-00961-s001.zip › ijms-2786751-supplementary.pdf]

CORRELATION BETWEEN INFLAMMASOMES AND MICROBIOTA IN PERI-IMPLANTITIS

SUPPLEMENTARY TABLES

Supplementary Table S1: Correlation between clinical and histological and immunohistochemical variables. Values indicate Spearman's rho correlation coefficient (p value).

	**Infiltrate Extension**	**AIM2 in lamina propria**	**AIM2 in epithelium**	**NLRP3 in lamina propria**	**NLRP3 in epithelium**	**Caspase1 in lamina propria**	**Caspase1 in epithelium**	**IL1b in lamina propria**	**IL1b in epithelium**
**Average PD**	-0.164 (p=0.386)	0.412 (p=0.027)	-0.145 (p=0.452)	0.488 (p=0.011)	-0.085 (p=0.685)	0.226 (p=0.248)	0.268 (p=0.177)	0.377 (p=0.057)	0.259 (p=0.221)
**Infiltrate Extension**	-	0.392 (p=0.035)	0.120 (p=0.534)	0.098 (p=0.633)	0.093 (p=0.660)	0.245 (p=0.209)	0.281 (p=0.155)	0.173 (p=0.397)	0.076 (p=0.722)
**AIM2 in lamina propria**	-	-	0.204 (p=0.289)	0.571 (p=0.003)	-0.159 (p=0.457)	0.475 (p=0.012)	0.260 (p=0.199)	0.315 (p=0.117)	0.170 (p=0.426)
**AIM2 in epithelium**	-	-	-	0.209 (p=0.317)	0.111 (p=0.606)	-0.067 (p=0.740)	0.037 (p=0.858)	-0.068 (p=0.743)	0.536 (p=0.007)
**NLRP3 in lamina propria**	-	-	-	-	0.244 (p=0.239)	0.277 (p=0.180)	0.075 (p=0.728)	-0.073 (p=0.733)	-0.112 (p=0.620)
**NLRP3 in epithelium**	-	-	-	-	-	0.066 (p=0.758)	0.171 (p=0.425)	-0.373 (p=0.079)	-0.062 (p=0.782)
**Caspase1 in lamina propria**	-	-	-	-	-	-	0.564 (p=0.002)	0.440 (p=0.028)	-0.090 (p=0.684)
**Caspase1 in epithelium**	-	-	-	-	-	-	-	0.412 (p=0.045)	0.442 (p=0.035)
**IL1b in lamina propria**	-	-	-	-	-	-	-	-	0.410 (p=0.047)
**IL1b in epithelium**	-	-	-	-	-	-	-	-	-

Supplementary Table S2: Diversity indexes.

Index	Value	Lower confidence interval (95%)	Upper confidence interval (95%)
**Chao**	715.84 (234.32)	510.64 (174.16)	1068.90 (326.84)
**Inverse Simpson**	10.84 (5.34)	10.00 (4.91)	11.83 (5.86)
**Shannon**	3.26 (0.48)	3.18 (0.48)	3.34 (0.48)
**Pielou**	0.61 (0.07)	-	-
